# A global optimization algorithm for protein surface alignment

**DOI:** 10.1186/1471-2105-11-488

**Published:** 2010-09-29

**Authors:** Paola Bertolazzi, Concettina Guerra, Giampaolo Liuzzi

**Affiliations:** 1 Istituto di Analisi dei Sistemi ed Informatica "A. Ruberti", Consiglio Nazionale delle Ricerche, Viale Manzoni, 30, 00185 Rome, Italy; 2 Dipartimento di Ingegneria Informatica, Universitá di Padova, Via Gradenigo, 6a, 35100 Padova, Italy; 3 College of Computing, Georgia Institute of Technology, Atlantic Drive, 801, 30332-0280 Atlanta (GA), USA

## Abstract

**Background:**

A relevant problem in drug design is the comparison and recognition of protein binding sites. Binding sites recognition is generally based on geometry often combined with physico-chemical properties of the site since the conformation, size and chemical composition of the protein surface are all relevant for the interaction with a specific ligand. Several matching strategies have been designed for the recognition of protein-ligand binding sites and of protein-protein interfaces but the problem cannot be considered solved.

**Results:**

In this paper we propose a new method for local structural alignment of protein surfaces based on continuous global optimization techniques. Given the three-dimensional structures of two proteins, the method finds the isometric transformation (rotation plus translation) that best superimposes active regions of two structures. We draw our inspiration from the well-known Iterative Closest Point (ICP) method for three-dimensional (3D) shapes registration. Our main contribution is in the adoption of a controlled random search as a more efficient global optimization approach along with a new dissimilarity measure. The reported computational experience and comparison show viability of the proposed approach.

**Conclusions:**

Our method performs well to detect similarity in binding sites when this in fact exists. In the future we plan to do a more comprehensive evaluation of the method by considering large datasets of non-redundant proteins and applying a clustering technique to the results of all comparisons to classify binding sites.

## Background

The function of a protein typically depends on the structure of specific binding sites located at the surface of the protein where the interaction with a ligand takes place. The identification of protein binding sites, their classification and analysis is of much interest for drug design and treatment of diseases. Binding sites recognition is generally based on geometry often combined with physico-chemical properties of the site since the conformation, size and chemical composition of the protein surface are all relevant for the interaction with a specific ligand.

In this paper we address the problem of optimally aligning protein surfaces, i.e. of finding atom pairs on two protein surfaces that occupy spatially equivalent positions. Our computational method integrates geometry with chemical properties of the matched atoms. It can be applied to the comparison of binding sites as well as of any other surface patches, such as cavities, that may be of interest.

Although the literature in protein surface alignment is not as vast as the one on complete structure or fold alignment, nevertheless several matching strategies have been designed for the recognition of protein-ligand binding sites and of protein-protein interfaces. They include hashing techniques [1,2], graph theoretic methods [3-6], descriptors based on moments [7] and moment invariants [8], shape descriptors such as spin images [9-11]. A few web servers have recently become available [12-16].

Most of the proposed methods require the solution to a 3D matching problem which is a well-studied problem also in computer vision and robotics. It can be formulated as follows: given two sets *A *and *B *of points, find two possibly large subsets *A*' of *A *and *B*' of *B *with high degree of *similarity*. There are various ways of defining the similarity between two point sets in 3D space leading to the proposal of different distance functions and associated algorithms; they include the root mean square distance, the closest point distance [17], the Hausdorff distance [18], the bottleneck distance [19].

An important aspect of the matching is the choice of a suitable surface representation; in the literature common ways of representing a surface are Connolly's representation [20], alpha-shapes [21] and pseudo-vertices [2]. In our approach, we represent the surface as a cloud of points, each corresponding to a surface atom. Thus, the protein surface alignment problem is the same as the aforementioned 3D matching problem.

One possible way to solve the surface alignment problem is by using the well-known Iterative Closest Point (ICP) algorithm [17] from which we draw our inspiration. The ICP algorithm, originally introduced in the area of computer vision for image registration, has been used in bioinformatics [22] for the alignment of complete protein structures. Indeed, we take a similar approach for surface alignment, namely we search for the isometric transformation which best superimposes two given protein structures.

Our main contribution is in the adoption of a different, more efficient global optimization approach along with a new dissimilarity measure. The global optimization algorithm we design belongs to the class of controlled random search methods [23-25]. These methods, although heuristic in nature, are very efficient and reliable for the global minimization of nonlinear multivariate functions of several variables. In the past years, controlled random search algorithms have been successfully used to solve many real world problems, see for instance [26-31]. The dissimilarity measure we propose is based on the solution to an "*Asymmetric Assignment Problem*" on a bipartite graph associated to the matching problem. Our method is capable of generating very accurate local alignments. We benchmark it on various sets of protein structures from the PDB [32], and compare its performance with that MolLoc [12].

## Notations and Assumptions

In this section we introduce some notations and assumptions that will be used throughout the paper. Given two protein structures P and Q, let us denote by *P *and *Q *the two finite sets of points corresponding to the atoms of the active sites of the two structures P and Q, respectively. We let

$$\begin{array}{ccc} n=|P| & \text{and} & m=|Q| \end{array}$$

and assume, without loss of generality, that *n *≤ *m*. The set *P *is conventionally representative of a query shape while *Q *defines a reference model shape.

An isometric transformation in three-dimensional space can be defined by a unit quaternion *a*_*r *_= (*a*_0_, *a*_1_, *a*_2_, *a*_3_)^⊤ ^∈ ℜ^4 ^(||*a_r_*|| = 1) and by a translation vector *a_t _*∈ℜ^3^. Let $a^{⊤}=(a_{r}^{⊤}a_{t}^{⊤})$ be the transformation defining vector and denote by *T_a _*the corresponding transformation, so that

$$y=T_{\alpha }(x)=R(a_{r})x+a_{t}$$

for every *x *∈ ℜ^3^, where *R *(*a_r_*) is the rotation matrix defined by the unit quaternion *a_r _*as follows:

$$R(a_{r})=\left(\text{​}\text{​}\text{​}\begin{array}{ccc} R_{11} & R_{12} & R_{13}\\ R_{12} & R_{22} & R_{23}\\ R_{13} & R_{23} & R_{33} \end{array}\text{​}\text{​}\text{​}\right)$$

where

$$\begin{array}{l} R_{11}=a_{0}^{2}+a_{1}^{2}-a_{2}^{2}-a_{3}^{2},\\ R_{12}=2(a_{1}a_{2}-a_{0}a_{3}),\\ R_{13}=2(a_{1}a_{3}+a_{0}a_{2}),\\ R_{22}=a_{0}^{2}+a_{2}^{2}-a_{1}^{2}-a_{3}^{2},\\ R_{23}=2(a_{2}a_{3}-a_{0}a_{1}),\\ R_{33}=a_{0}^{2}+a_{3}^{2}-a_{1}^{2}-a_{2}^{2} \end{array}$$

Let Θ ⊂ ℜ^7 ^be the set of all vectors *a *∈ ℜ^7 ^defining an isometric transformation in ℜ^3^. Given a transformation vector *a *∈ Θ, let *T_a _*(*P*) = *P_a _*denote the set of points obtained by applying the transformation *T_a _*to every point of *P*, that is

$$T_{a}(P)=P_{a}=\{y:y=R(a_{r})p+a_{t},\;\forall \;p\in P\}.$$

Let: *ψ: P *→ *Q *denote a point to point mapping that associates to every point of *P *a point of *Q*. Since, as assumed above, *P *and *Q *are finite sets, the class Ψ of all mappings *ψ *has finite cardinality given by |Ψ| = *m^n^*.

Let *ψ *∈ Ψ be a given mapping and *a *be a vector defining an isometric transformation, then the mean square error function between *P *and *Q *is the following

(1) $$f(\psi ,a)=\frac{1}{n}\sum_{p\in P}\|\psi (p)-R(a_{r})p-a_{t}{\|}^{2}.$$

The surface alignment problem consists in finding a mapping ψ* ∈ Ψ of points in *P *to points in *Q *and an isometric transformation a* such that

f(ψ*,a*)≤f(ψ,a),

for all *ψ *∈ Ψ and *a *∈ Θ.

The problem can also be formulated in terms of the following definition of an assignment. A function *φ *∈ Ψ is an *assignment *from *P *to *Q *if, by definition, it is injective, that is for every *p*_1_, *p*_2 _∈ *P*, *p*_1 _≠ *p*_2 _implies *φ*(*p*_1_) ≠ *φ *(*p*_2_).

Let us denote by Φ ⊆ Ψ the class of all possible assignments from *P *to *Q*. Obviously, since *P *and *Q *are finite sets, Φ is finite as well and its cardinality is |Φ| = *m *(*m *- 1) ... (*m *- *n *+ 1).

## Results

### Algorithm

A well-known algorithm for shape alignment is the Iterative Closest Point Algorithm [17]. This algorithm stems from the idea that, once a mapping $\bar{\psi }$ ∈ Ψ is fixed, it is possible to compute the isometric transformation *a *∈ Θ that minimizes the function *f*($\bar{\psi }$, *a*) (a closed-form expression for *a*($\bar{\psi }$) has been given in [33] where we refer the interested reader for the relevant details). Let *a*($\bar{\psi }$) be the minimizer of *f*($\bar{\psi }$, *a*), that is

$$a(\bar{\psi })=\text{arg}\underset{a\in \Theta }{\min}f(\bar{\psi },a).$$

Hence, the problem implicitly considered by the ICP Algorithm is the following two-level optimization problem

(2) $$\begin{array}{l} \min_{\psi }f(\psi ,a)\\ s.t.\psi \in \Psi \\ a=\text{arg}\underset{a\in \Theta }{\min}f(\psi ,a). \end{array}$$

As it is stated in [17], where ICP has been originally proposed, the method converges to a solution which is a local minimum of the two-level Problem (2). Futher, in [17] it has been shown that the final transformation $\bar{a}$ and mapping $\bar{\psi }$ obtained by Algorithm ICP heavily depend on the initial relative positioning of sets *P *and *Q*.

In this section we discuss the use of a continuous global optimization algorithm for the solution of the shape alignment problem. To this aim, it is necessary to reformulate the shape alignment problem in a complementary way with respect to Problem (2). More in particular, the inner-level problem becomes the one defining the mapping function *ψ *(instead of the transformation *a*) once the transformation vector *a *∈ Θ is fixed in the outer level.

Namely, we consider the following two-level optimization problem

(3) $$\begin{array}{l} \min_{a}f(\psi ,a)\\ s.t.a\in \Theta \\ \psi =\arg\underset{\psi \in \Psi }{\min}f(\psi ,a). \end{array}$$

Problem (3) can be reduced to a one-level optimization problem by considering that for every vector *a *∈ Θ, the inner-level problem of (3) admits a globally optimal solution, which we denote by

(4) $$\psi (a)=\text{arg}\underset{\psi \in \Psi }{\min}f(\psi ,a)$$

and represents the closest point mapping. Hence, Problem (3) can be equivalently stated as

(5) $$\underset{a\in \Theta }{\min}\;g(a)$$

where *g*(*a*) = *f*(*ψ*(*a*), *a*). Every global solution a* of (5) is, by definition, a solution such that *f *(*ψ *(a*), a*) ≤ *f *(*ψ *(*a*), *a*), for all *a *∈ Θ.

Observe that the computation of function *g *requires the computation of the optimal mapping *ψ *(*a*), that is, the solution to Problem (4). This latter problem can be solved with a time complexity *O*(*nm*) in the worst case [17] which can be relevant for *n *and *m *large. Moreover, due to its definition, *g*(*a*) is a non-smooth (Lipschitz) continuous function and its derivatives are not available. Indeed, for the minimization of function *g*(*a*) we can neither directly use its derivatives nor approximate them through finite differences since this would require too much time and produce numerical derivatives which are unreliable because of the non-smoothness of function *g*.

On the basis of the above observation we propose the use of a controlled random search method for the solution to Problem (5). In the following we briefly recall the global optimization algorithm that we use and which was originally proposed in [25] and successively improved in [24]. It is a population based algorithm in the sense that, through-out the entire optimization process, a population of points is maintained and iteratively updated in such a way that they cluster around the global minima of the objective function. Roughly speaking, the method is composed of two distinct and consecutive phases: a global phase and a local phase. During the global phase an initial population of points (defining roto-translations in three-dimensional space) is generated by randomly sampling a sufficiently large set of points over some feasible domain. Then, at every iteration of the local phase, a new point is generated and the population is updated if this new point improves on the worst point of the population. More in details, the algorithm can be described by the following steps.

1. **initialization** Let *N *= 6 and choose an integer *M *≫ *N*. The objective function is sampled on a set *S *of *M *points *a *∈ ℜ^7 ^randomly chosen within the feasible domain Θ strictly containing the global minimizer.

2. **stopping criterion** If the maximum and minimum values of the objective function over *S *are sufficiently close to each other, namely

$$g_{\max}-g_{\min}<\epsilon ,$$

where

$$\begin{array}{cc} g_{\max}=\underset{a\in S}{\max}g(a), & g_{\min}=\underset{a\in S}{\min}g(a) \end{array}$$

then STOP.

3. **search phase***N *+1 points are randomly chosen in the set *S*. Then,

(a) the *weighted *centroid *a_c _*of the *N *+ 1 points is computed;

(b) the new trial point *ã *is computed by doing a *weighted reflection *of the centroid onto the worst point among the selected *N *+ 1 points.

Namely, let *a*^† ^be the worst point, then

$$\begin{array}{ccc} \overset{ˇ}{a}=(1+\alpha )a_{c}-\alpha a^{†}, & \text{and} & \tilde{a}=W\overset{ˇ}{a} \end{array}$$

where *α *∈ (0,1) is a reflection parameter and

$$\begin{array}{l} W=\left(\begin{array}{ccccccc} 1/\xi & 0 & \ldots & \ldots & \ldots & \ldots & 0\\ 0 & 1/\xi & 0 & \ldots & \ldots & \ldots & 0\\ 0 & 0 & 1/\xi & 0 & \ldots & \ldots & 0\\ 0 & \ldots & 0 & 1/\xi & 0 & 0 & 0\\ 0 & \ldots & \ldots & 0 & 1 & 0 & 0\\ 0 & \ldots & \ldots & \ldots & 0 & 1 & 0\\ 0 & \ldots & \ldots & \ldots & \ldots & 0 & 1 \end{array}\right),\\ \xi =||{\left({\overset{ˇ}{a}}_{0}{\overset{ˇ}{a}}_{1}{\overset{ˇ}{a}}_{2}{\overset{ˇ}{a}}_{3}\right)}^{⊤}|| \end{array}$$

The normalization matrix *W *is introduced to ensure that the first four components of the resulting point represent a unit quaternion (i.e. a rotation).

The parameter α is iteratively updated during the optimization process [24] in such a way that its value tends to zero as the iteration count increases and the difference *g*_max _- *g*_min _decreases.

4. **updating phase** If the objective function value on the new point *ã *improves on the maximum function value over *S*, then the set *S *is updated by adding the new point and discarding the worst one. Otherwise the set *S *is left unchanged and the new point is discarded. The algorithm continues iterating through steps 2-4.

The algorithm starts by randomly choosing *M *≫ *N *points over the feasible set Θ. In the literature, a typically accepted value of *M *is 25*N *[25,29]. This value is able to convey to the algorithm sufficient ability to find the global minimum point without excessively slowing down the convergence.

### A new dissimilarity measure

In this section we propose a new dissimilarity measure between two given sets of points of two proteins. This measure is based on a distance other than the closest point distance.

In particular, it can be noted that, using the closest point distance, it can happen that different points of set *T_a_*(*P*) are mapped to the same point of set *Q*. This, in turn can yield a distance value which is small just because many points are all mapped to the same closest point.

In order to avoid this undesirable effect, we consider the function *f*(*φ*, *a*) defined in (1) where *φ *∈ Φ is an assignment function and let, for every *a*, *φ *(*a*) be a global solution to problem

(6) $$\underset{\varphi \in \Phi }{\min}f(\varphi ,a).$$

Problem (6) can be formulated as a 0, 1-optimization problem and is, indeed, the combinatorial optimization problem known as the *Asymmetric Assignment Problem *(AAP).

In particular, let *G *(*P*, *Q*, *E*), *E *⊂ *P *× *Q*, be the bipartite directed graph characterized by the two sets of nodes *P *and *Q *and by the edges between all pairs of nodes, one of *P *and the other of *Q*. Then, for every pair *e *= (*p*, *q*) ∈ *E*, define

$$c_{e}={\left\|q-T_{a}(p)\right\|}^{2}.$$

Let *s *∈ {0, 1}^|*E*| ^be the edge incidence vector and consider the following minimum cost assignment problem

(7) $$\begin{array}{l} \underset{s}{\min}c^{⊤}s\\ \sum_{e\in \delta^{+}(p)}s_{e}=1,\;\;\forall p\in P\\ \sum_{e\in \delta^{-}(q)}s_{e}\leq 1,\;\;\forall q\in Q\\ s\in {\left\{0,1\right\}}^{\left|E\right|}, \end{array}$$

where, *δ*^+ ^(*p*) and *δ*^- ^(*q*) are the sets of edges leaving node *p *and, respectively, entering in node *q*.

Note that the constraints of Problem (7) require each node *p *∈ *P *to be assigned to exactly one node *q *∈ *Q *and each node *q *∈ *Q *to be assigned to at most one node *p *∈ *P*, which is why Problem (7) is known as *Asymmetric Assignment Problem*.

Clearly, it is

$$f(\varphi \;(a),a)=c^{⊤}s*$$

where s* is the optimal solution to Problem (7).

Problem (7), and hence (6), can be solved very efficiently by *ad-hoc *codes that have time complexity $O(\sqrt{nm}\log(nC(T_{a})))$ where $C\left(T_{a}\right)={\text{max}}_{p\in P,q\in Q}\left\{\left||q–T_{a}\left(p\right)|\right|\right\},$ see for instance [34].

We are now able to define our new dissimilarity mea-sure, that we call *matching distance*.

**Definition 1 ***Given an isometric transformation a *∈ Θ *and two distinct sets of points P and Q, the matching distance between T_a_*(*P *) *and Q is given by f*(*φ*(*a*), *a*).

Reasoning as in the preceding section, we can now search for a global solution to problem (5) where now *g*(*a*) = *f*(*φ*(*a*), *a*).

### Integration of physico-chemical properties

Up to this point, the discussed approach is based on geometry only. However, as is well known in biology, there are other properties that affect the binding of molecules. For instance, electrostatic as well as hydrophobic-hydrophilic properties play an important role in protein-protein and protein-ligand interactions. Thus, we consider a variant of our approach in which we integrate physico-chemical properties. Specifically in the graph *G*(*P*, *Q*, *E*) we assume that the edge *e *= (*p*, *q*) is present only if the two atoms *p *∈ *P *and *q *∈ *Q *have the same physico-chemical properties. According to [35], we say that *p *and *q *have the same physico-chemical properties if they are both Acceptor (ACC), Donor (DO), Acceptor/Donor (AD), Aliphatic (ALI) or Aromatic (PI). Furthermore, we assume that, for every *p *∈ *P *at least a node *q *∈ *Q *exists such that (*p*, *q*) ∈ *E*.

### Testing

We applied our method, referred to as Continuous Optimization (CO) method in the following, to the comparison of binding sites of proteins. We integrated physico-chemical properties in our method, as discussed in the previous section. The structures of the proteins in complex with specific ligands are taken from the PDB [32]. The binding sites are extracted by a simple algorithm that finds all protein atoms within a certain distance (4.0 Å) from an atom of the ligand. We run our algorithm on pairs of binding sites producing in output the list of matched atoms on the two binding sites, the rigid transformation that best superimposes them, and the RMSD after superposition.

We benchmarked CO on a dataset of 100 proteins in complex with 9 ligands that differ in chemical composition as well as in size and shape. The results of all-to-all pairwise comparisons are visualized by means of a distance matrix and by the ROC curves. The goal is to evaluate the ability of CO in assigning a binding site to the correct group of proteins, i.e. those binding the same ligand.

We then present more detailed results on a set of 19 binding sites of proteins in complex with the ligand ATP with the goal of judging the quality of the alignments. For each comparison we report the number of aligned atoms as well as their RMSD after superposition. The results on this dataset are compared with those of another method, MolLOC [12], which derives the same two measures, i.e. number of aligned atoms and RMSD.

### Classification of proteins according to their bound ligand

In the first experiment we perform all-to-all comparisons on a dataset of 100 proteins in complex with one of 9 ligands: AMP, ATP, FAD, FMN, GLC, HEME, NAD, PO4, and Steroid. This dataset was used in [36] for an analysis of shape variation in protein binding sites. The proteins were carefully selected, with a number of criteria, so that the dataset is non-redundant and the binding sites are not evolutionary related.

**Figure 1:**
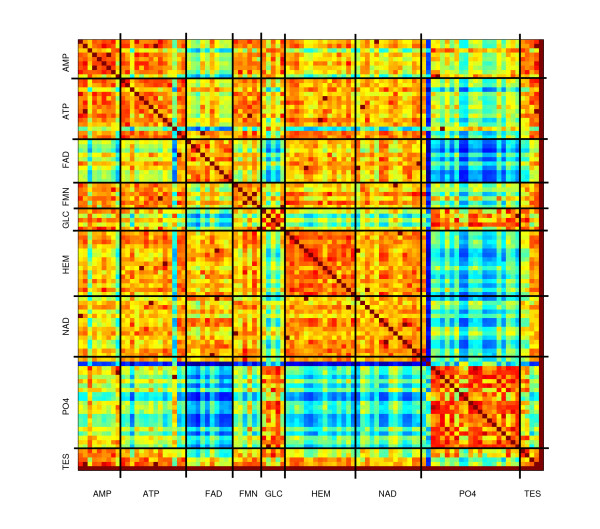
**Distance matrix**. The matrix shows the results of all-to-all comparisons. The 9 ligands are indicated along the rows and columns and the proteins binding each ligand are grouped together. The grid of horizontal and vertical black lines separates different groups of proteins. The matrix is color-coded from 0 (blue) to 1 (red), with red corresponding to high number of aligned atoms and therefore high similarity in the shape of the binding sites and blue to the lowest degree of similarity.

The results of all-to-all comparisons are illustrated by means of the distance matrix of Figure 1. An entry of the matrix corresponds to a protein pair and contains a value related to the number of aligned atoms of the binding sites of the pair. Namely, in the matrix we report

$$\text{2}\left(\frac{\text{num}\text{.}\;\text{aligned}\;\text{atoms}}{n+m}\right)$$

where *n *and *m *are the numbers of atoms of the two binding sites. The proteins are listed along the rows and columns of the matrix so that proteins binding the same ligand are grouped together. Horizontal and vertical black lines on the matrix separate different groups of proteins. The matrix is color-coded from 0 to 1, with red corresponding to high number of aligned atoms and therefore high similarity in the shape of the binding sites and blue to the lowest degree of similarity. A good classification of sites based on bound ligands implies the presence of mostly red areas around the main diagonal, corresponding to pairwise comparisons within the same group of proteins, i.e. in complex with one specific ligand. This can be in fact observed in the image matrix although with different degrees for the different groups of proteins. As it is known [36], ligand PO4 tends to be rigid, exhibiting little conformational variability in the binding. Not surprisingly, the corresponding area is the one showing the highest degree of similarity. The method CO appears to perform well also in distinguishing the PO4 group from any other group, as PO4 binding sites are more similar to themselves than to binding sites of other groups. Similar considerations apply to steroid and GLC. A good performance is also obtained for the HEME group, although the discriminating power with the NAD group is not clear. As noted in [37], ligand ATP has great variation in its conformation when binding different proteins: it can be in an extended conformation or in a compact one, resulting in different sizes and shapes of the binding regions. This is reflected in our experiments, as can be seen from the distance matrix where blue or green areas are present.

**Figure 2:**
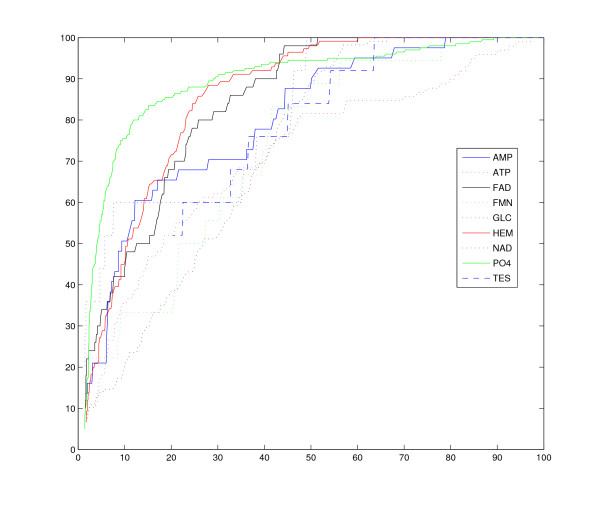
**ROC curves**. The curves reported in the figure show the fraction of true positives or correct answers vs. the fraction of false positives for all positions of the ranked solutions. Each curve in the figure shows the average values obtained on all query proteins of a group. As expected, the curve corresponding to PO4 (in green) deviates the most from the diagonal line being the closest to the top-left corner of the ROC square. Thus CO has a good success in predicting a PO4 binding site. By contrast, the worst performance is achieved for NAD binding proteins with the associated curve in dotted black.

An important aspect of an alignment method is its ability to retrieve, for a given query binding site, those proteins of the dataset binding the same ligand. To evaluate CO in this task we resort to ROC curves. The results of the comparisons of the query with all other proteins are ranked from the best to the worst in terms of the number of aligned atoms. A pairwise comparison in the ranked list is considered correct or true positive if the other protein of the pair binds the same ligand as the query. The results are summarized by the receiver operating characteristic (ROC) curves in Figure 2 that display the fraction of true positives or correct answers vs. the fraction of false positives for all positions of the ranked solutions. The best possible prediction results would yield a curve through the point in the upper left corner or coordinate (0, 1) of the ROC space. A completely random guess would give a point along a diagonal line from the left bottom to the top right corners. We repeated this experiment with each protein of the dataset as query. Each curve in Figure 2 shows the average values obtained on all query proteins of a group. As expected, the curve corresponding to PO4 (in green) deviates the most from the diagonal line being the closest to the top-left corner of the ROC square. Thus CO has a good success in predicting a PO4 binding site. By contrast, the worst performance is achieved for NAD binding proteins with the associated curve in dotted black.

From the above sets of experiments we can conclude that CO has a good accuracy in the retrieval of similarity information: for a given query binding site the highest scoring solutions are generally the binding sites of the dataset in complex with the same ligand as the query. Furthermore, when a good similarity in the binding is expected because of the relative rigidity of the ligands, CO is able to capture such a similarity, as shown in the distance matrix.

### Comparing CO with other alignment methods on ATP binding sites

Several studies have been conducted to evaluate and compare different methods for determining the structural similarity of proteins. For instance, a comprehensive assessment of structural alignment methods is presented in [38] where six publicly available programs are evaluated on almost 9 million pairs of proteins. However, a similar large-scale experiment is not available for the related problems of aligning protein surfaces and binding sites, despite the growing number of methods and web servers available. There are several factors that contribute to the difficulty of the comparison. First, different methods solve different instances of the matching problem: some methods compare binding sites, while others recognize binding sites in cavities or even entire surfaces. Second, the methods differ in the input representations and scoring functions. For instance, in CO the input points are the atom centers, in Multibind a reduced set of points, the pseudo-centers. In [36] the points are the spherical sample points derived from the atomic coordinates. MolLoc, on the other hand, uses Connolly's [39] points and a richer surface representation based on local shape descriptors of surface points. As for the scoring function, although most methods produce the RMSD of the superimposed structures, some methods have a different native scoring function that cannot be easily derived by other methods.

As a comprehensive evaluation of all the techniques is beyond the scope of this paper, only MOlLoc will be considered in comparison with CO. The reason for choosing MolLoc is that both methods judge the quality of the alignments by the number of aligned atoms and their RMSD after superposition. Such measures are available at MolLOC website. As Multibind does not report the RMSD of two aligned structures at its website it will not be considered here. Moreover, the method in [36], based on spherical harmonics and benchmarked on the same dataset of 100 proteins, is not used in our evaluation because it computes a measure of similarity of two shapes without an alignment.

As observed in [38], although the ROC curves are a valid tool for assessing the quality of a classification approach they are often of limited value in comparing different methods; in fact such curves take into account only the ranking of the alignments not their quality. For this reason, since we want to assess the quality of the alignments we choose the geometric measure SAS [38,40,41]. Clearly, a better match has a higher number of aligned atoms and smaller RMSD. Since the two measures are not independent SAS combines them into a single expression: SAS = (RMSD × 100)/(num. aligned atoms).

We run both programs on the set of 19 proteins used in [42] for a related although different problem, that is binding site recognition within a cavity. The proteins all bind ligand ATP and are from different families according to the structural classification SCOP [43].

We performed pairwise comparisons of the active site of the Catalytic Subunit of cAMP- dependent Protein-Kinase (pdb code 1atp, chain E) with each of the remaining proteins of the input data set. Of the set of proteins only three belong to the same SCOP family as 1atp, namely 1phk, 1csn and 1hck. In Table 1 for each comparison we report the number of aligned atoms along with the RMSD obtained by CO (columns 3-4) and MolLoc (colums 6-7). The entries of the table are listed and ranked according to the number of corresponding atoms obtained by CO (column 3). We observe (see Table 1) that both methods correctly rank at the top three positions the proteins in the same family as 1atp, that is 1phk, 1hck and 1csn. Furthermore for the same three proteins the RMSD is typically very low (approx. 1.5 Å). Lower scores are obtained for distantly related proteins, as for instance 1g5t. The table also reports the SAS measure for CO (column 5) and MolLoc (column 8) and their average at the bottom of the same two columns. As a lower SAS value indicates a better match, it follows that CO on average achieves a better quality than MolLoc with respect to this measure.

**Table 1:** Comparison of CO with MolLoc.

		**CO**			**MolLoc**		
**Rank**	**Protein Pair**	**N. corresp atoms**	**RMSD**	**SAS**	**N. corresp atoms**	**RMSD**	**SAS**

1	atpE-1hck	62	1.2	1.94	45	1.3	2.89
2	1atpE-1phk	57	0.91	1.6	63	0.9	1.43
3	1atpE-1csn	50	1.18	2.36	55	0.9	1.64
4	1atpE-1nsf	34	2.11	6.21	11	1.4	12.73
5	1atpE-1j7k	25	1.81	7.24	25	1.6	6.4
6	1atpE-1e8xA	24	1.74	7.25	20	1.7	8.5
7	1atpE-1f9aC	21	2.17	10.33	18	1.6	8.89
8	1atpE-1kay	20	1.9	9.5	8	1.7	21.25
9	1atpE-1yag	20	1.92	9.6	17	1.6	9.41
10	1atpE-1a82	19	2.02	10.63	13	1.9	14.62
11	1atpE-1jjv	18	1.76	9.78	10	1.8	18
12	1atpE-1gn8A	17	2.37	13.94	14	1.6	11.43
13	1atpE-1b8aA	16	2.05	12.81	10	2	20
14	1atpE-1mjhA	16	2.28	14.25	14	1.9	13.57
15	1atpE-1e2q	15	1.39	9.27	5	1.8	36
16	1atpE-1kp2A	13	1.51	11.62	15	1.9	12.67
17	1atpE-1ayl	12	1.21	10.08	16	2	12.5
18	1atpE-1g5t	7	2.26	32.29	8	1.6	20
			avg.SAS	10.04		avg.SAS	12.88

Pairwise comparisons of the binding site of protein 1atp with other 18 proteins all binding ATP (columns 2). The results of CO (columns 3-5) and MolLoc (columns 6-8). For a de_nition of SAS see the text. The comparisons are ranked based on the number of corresponding atoms in CO (column 3).

**Figure 3:**
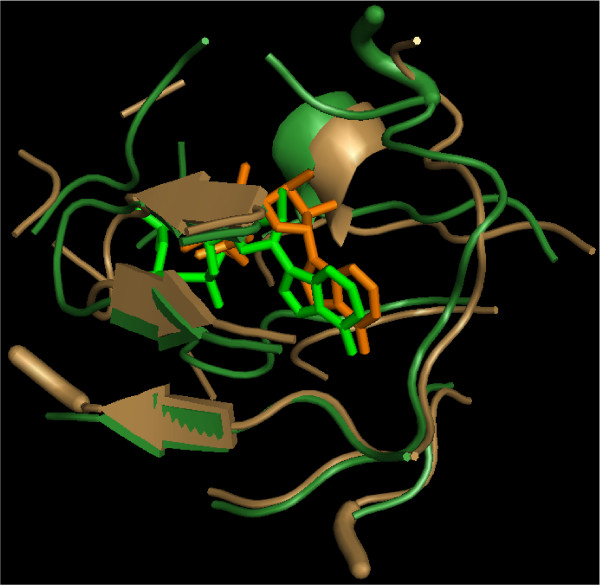
**Example of a computed superimposition**. Comparison of CO with MolLoc. Pairwise comparisons of the binding site of protein 1atp with other 18 proteins all binding ATP (columns 2). The results of CO (columns 3-5) and MolLoc (columns 6-8). For a definition of SAS see the text. The comparisons are ranked based on the number of corresponding atoms in CO (column 3).

Figure 3 shows an example superimposition of the binding sites of ligand ATP of proteins 1atp and 1hck after the computed rototraslation is applied.

We conclude this section by reporting that the execution times of CO on average on all 18 pair-wise comparisons considered in this experiment was 14.06 s for a total of 253.1 s on an Intel Pentium IV processor running at 2.66Ghz with 1Gb main memory. As we mentioned before, the low computational complexity of our proposed approach is one of the key points of our design. We do not report the execution times of MolLoc since they are not available from the web server interface.

## Discussion and Conclusions

The main challenge for a method that compares and classifies binding sites is to be able to cluster the binding sites in groups according to the type of ligands they bind while at the same time allowing some conformational variability within the same group, as is often observed for binding sites of different proteins complexed with the same ligand. The difficulty arises because of the variety of ways in which a ligand can bind proteins. Although we expect a computational method to be able to distinguish among different types of ligands relatively well, there are obviously cases when only experimental methods can determine the binding affinity of two molecules.

Our proposed method, CO, performs well to detect similarity in binding sites when this in fact exists. In the future we plan to do a more comprehensive evaluation of the method by considering large datasets of non-redundant proteins and applying a clustering technique to the results of all comparisons to classify binding sites. A systematic evaluation of CO with other existing methods will be done through the introduction of a common scoring function that will overcome the problem that the available methods use native scoring functions difficult to export to other methods.

## Authors' contributions

All three authors participated in the design of the methodology and in set up of the experiments. GL carried out the implementation. All authors read and approved the final manuscript.
